# Treatment of a mutant *KRAS* lung cancer cell line with polyisoprenylated cysteinyl amide inhibitors activates the MAPK pathway, inhibits cell migration and induces apoptosis

**DOI:** 10.1371/journal.pone.0312563

**Published:** 2024-10-22

**Authors:** Matthew D. Gregory, Kweku Ofosu-Asante, Jassy Mary S. Lazarte, Pablo E. Puente, Nada Tawfeeq, Nadine Belony, Yong Huang, Ite A. Offringa, Nazarius S. Lamango

**Affiliations:** 1 Institute of Public Health, Florida A&M University College of Pharmacy Pharmaceutical Sciences, Tallahassee, FL, United States of America; 2 Keck School of Medicine, University of Southern California/Norris Comprehensive Cancer Center, Los Angeles, CA, United States of America; 3 University of Florida Department of Mechanical and Aerospace Engineering, Gainesville, FL, United States of America; Kyoto Prefectural University of Medicine, JAPAN

## Abstract

*KRAS* mutations are the most common oncogenic mutations in lung adenocarcinoma in Black Americans. Polyisoprenylated Cysteinyl amide Inhibitors (PCAIs) constitute a group of potential cancer therapy agents that we designed to specifically disrupt and suppress hyperactive G-protein signaling, such as that caused by mutated RAS proteins. Here we determine the effects of PCAIs on the viability, G-protein levels, downstream mediators, and apoptosis-related proteins on the *KRAS*-mutated, Black American-derived lung adenocarcinoma cell line, NCI-H23. Of the 17 PCAIs tested, compounds NSL-YHJ-2-27 and NSL-YHJ-2-46 showed the most potency with EC_50_ values of 2.7 and 3.3 μM, respectively. Western blotting was used to determine the effect of the PCAIs on the phosphorylation levels of MAPK pathway enzymes. After 48 h exposure to 5 μM of the PCAIs, NSL-YHJ-2-46, the MAPK proteins BRAF, MEK1/2, ERK1/2, and p90RSK were activated through phosphorylation by 90, 190, 150 and 120%, respectively. However, CRAF/RAF1 phosphorylation decreased by 40%, suggesting significant changes in the KRAS/MAPK signaling patterns. Furthermore, 5 μM of NSL-YHJ-2-27 depleted the singly polyisoprenylated monomeric G-proteins RAC 1/2/3 and CDC42 by 77 and 76%, respectively. The depletion of these key cytoskeletal proteins may account for the observed inhibition of cell migration and invasion, and spheroid invasion observed on exposure to NSL-YHJ-2-27 and NSL-YHJ-2-46. Treatment with 5 μM of NSL-YHJ-2-27 suppressed full-length inactive caspase 3 and 7 levels by 72 and 91%, respectively. An analysis of cells treated with the fluorescently labeled active caspase 3/7 irreversible inhibitor, CaspaTag^TM^ Caspase-3/7 *in situ* reagent revealed a 124% increase in active caspase at 3 μM over controls. These findings clearly show the direct effects of the PCAIs on the RAS signaling pathway. Given the profound increases observed in RPS6KA1/p90RSK phosphorylation, future work will involve a determination whether the proapoptotic isoforms of RPS6KA1/p90RSK are phosphorylated due to the PCAIs treatments. These results support the potential use of the PCAIs as targeted therapies against cancers with *KRAS* mutations.

## Introduction

Lung cancer is the second most common cancer and the major cause of cancer-related deaths of American men and women [1]. Black American men show the highest lung cancer incidence and mortality of all races/ethnicities, with an incidence of 77.4 per 100,000 and a mortality rate of 47.0 per 100,000 [1]. Mutated *KRAS* is the most common driver gene in lung adenocarcinoma, the most common histological subtype of lung cancer, in White and Black Americans alike [1]. While the presence of *KRAS* mutations may be similar in Blacks and Whites, other associated genetic factors may influence responses to various therapeutic agents. It is therefore imperative to gain a better understanding of cancer-driving alterations in the RAS pathway, particularly in Black Americans, and to develop new drugs targeting this pathway. The drugs should function in all genetic backgrounds, particularly in those of Black males.

**Fig 1:**
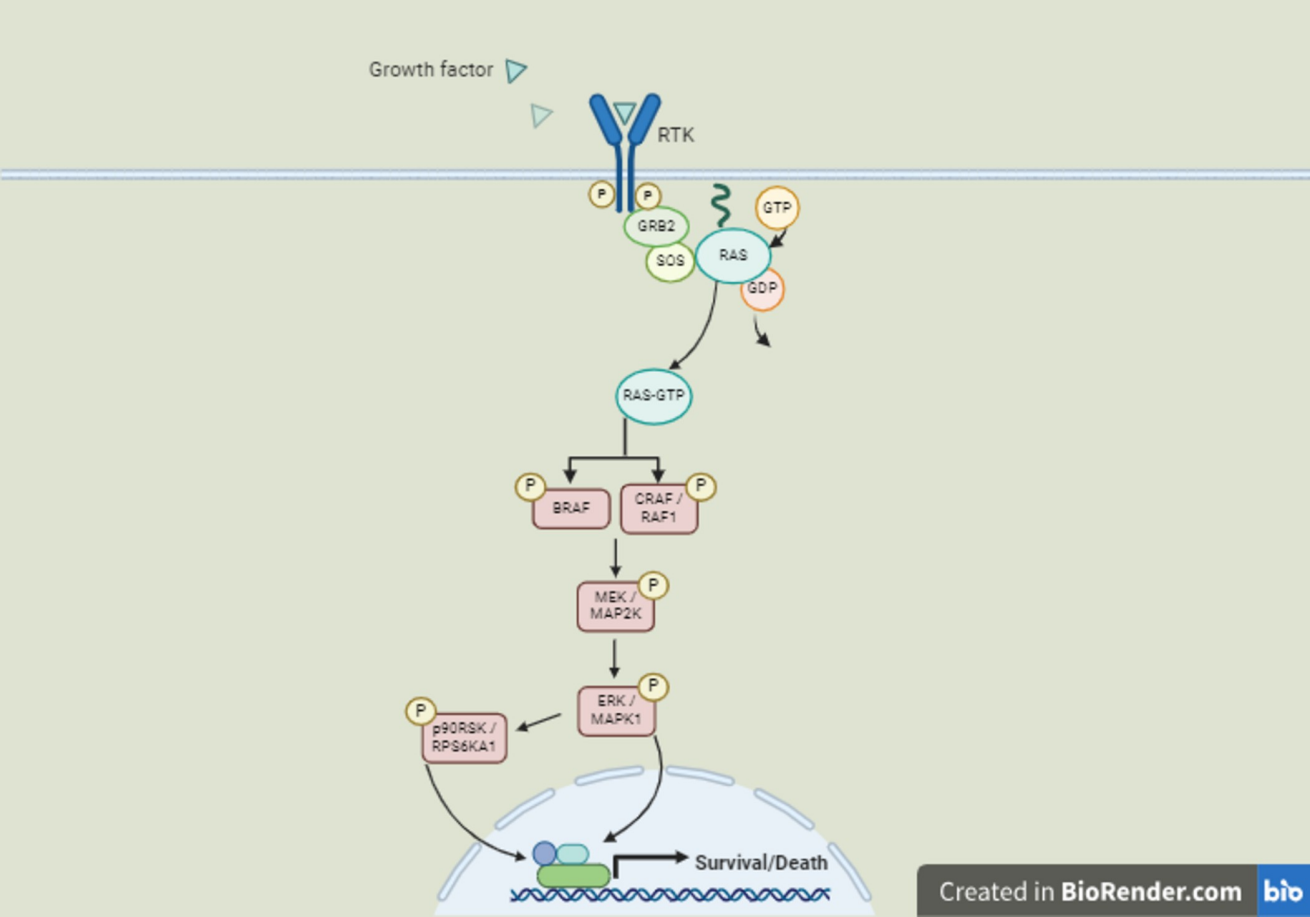
Schematic diagram of the RAS signaling pathway. Mediators whose cellular phosphorylation levels have so far been shown to be altered by PCAIs treatment are indicated by pink rectangles and previously developed drugs that target them by white rectangles. Created with BioRender. P indicates phosphorylation. RTK: Receptor tyrosine kinase.

Various biomolecular changes contribute to the dysregulation of the mitogen-activated protein kinase/extracellular signal-regulated kinase (MAPK/ERK) signaling pathway (Fig 1) in cancers. Epidermal growth factor (EGF) overexpression, EGF receptor (EGFR) overexpression and/or activating mutations of its intracellular kinase domains have been identified as drivers in various cancers, including lung cancer [2–4]. EGFR signals are passed on to rat sarcoma virus (RAS) proteins through such intermediates as growth factor receptor binding proteins and son-of-sevenless (SOS), a guanine nucleotide exchange factor (GEF) [5]. There are three *RAS* genes, Kirsten *RAS (KRAS*), neuroblastoma *RAS (NRAS)* and Harvey *RAS (HRAS)*, whose proteins relay the extracellular messages from the membrane-bound receptors to downstream mediators and effectors [6]. Activating mutations involving all the *RAS* genes have been detected in cancers and drive about 33% of human neoplasms [7]. *KRAS* mutations are by far the most common, accounting for 86% of the mutant *RAS* cases [8]. In Black and White Americans, *KRAS* mutations are found with similar frequencies [9]. RAS proteins belong to the superfamily of guanine nucleotide-binding proteins known as G-proteins [6]. They function as molecular switches, alternating between the active GTP-bound and the inactive GDP-bound states [10]. The active states extinguish their signaling through an intrinsic GTPase activity that is mediated by the GTPase-activating proteins (GAPs) [10]. Mutant oncogenic RAS proteins are dysregulated by loss of the GTPase activity, remaining constitutively in the active GTP-bound state that stimulates the phosphorylation-dependent activation of downstream mediators such as the rapidly accelerated fibrosarcoma (RAF) proteins, BRAF, RAF1 (CRAF), MAP2K1/2 (MAP ERK kinase/MAP kinase kinase/MEK1/2), MAPK1/3 (extracellular signal-regulated kinase/ERK1/2) and RPS6KA1 (p90 ribosomal protein S6 kinase/p90RSK) [11]. Cell survival and proliferation then ensue due to this RAS hyperactivity. Activating mutations of the genes encoding the kinases BRAF, RAF1, MAP2K1 and 2 and MAPK1/3 are also known cancer drivers [12].

Cancers driven by mutant RAS proteins have been considered undruggable until recently. The challenge has always been to design compounds that can competitively displace GTP from RAS given the cellular micromolar GTP concentrations and its picomolar dissociation constants from RAS proteins [13,14]. Consequently, earlier efforts to develop anti-RAS drugs centered on the synthesis of farnesylation inhibitors as it was observed that the C-terminal farnesyl cysteine is essential for RAS membrane localization and function [15]. However, success with this approach proved elusive as geranylgeranylation ensued to circumvent the effects of the farnesyl transferase inhibitors (FTIs) [16]. Furthermore, the FTIs were highly toxic [16]. An indirect and somewhat more successful approach to deal with the RAS conundrum was the development of kinase inhibitors targeting the downstream mediators of RAS signaling, RAF, MEK1/2 and ERK1/2 [17]. More recently, chemists are engaging in the design of small molecules that fit into tiny pockets on KRAS, targeting and chemically reacting with specific mutant RAS protein subsets to allosterically inhibit and disrupt oncogenic signaling [18,19]. These exciting new approaches that have resulted in clinically available drugs like Adagrasib and Sotorasib which are changing the prior narrative about mutant RAS proteins being undruggable.

Another targeted drug development approach that involves interfering with the RAS interactome derives from molecules initially designed to inhibit polyisoprenylated methylated protein methyl esterase/carboxylesterase 1 (PMPMEase/CES1), an enzyme that is overexpressed in various cancers [20–22]. Early prototypical irreversible PMPMEase inhibitors induced apoptosis in various cancer cell lines [23,24]. We then developed the polyisoprenylated cysteinyl amide inhibitors (PCAIs) as potential reversible inhibitors of PMPMEase enzyme. However, the PCAIs were poor PMPMEase inhibitors [20] but were found to be significantly more effective against cell viability, migration and invasion, angiogenesis, and induced retraction of filopodia and lamellipodia resulting in cell rounding [25–28]. These phenotypic observations were correlated with cellular such molecular changes as disruption of actin filaments, depletion of KRAS, cell division cycle 42 (CDC42), Rac family small GTPase 1 (RAC1), fascin and vinculin and disintegration of vinculin punctates [27,28], suggesting their potential to treat *KRAS*-mutated cancers. In this manuscript, we use lung adenocarcinoma cell line NCI-H23, derived from a Black male and carrying mutated *KRAS*, to study the effect of PCAIs on RAS pathway enzymes and signaling.

## Materials and methods

### Materials

Cell lines were obtained from American Type Culture Collection (ATCC, Manassas, VA). The identity of NCI-H23 cells was confirmed by DNA fingerprinting, and the presence of the *KRAS^G12C^* mutation was confirmed by localized sequencing, with NCI-H23 cells possessing a point mutation in codon 246 of the *p53* gene alongside the expression of PDGF A and B chains. The FITC Annexin V/ Dead Cell apoptosis kit (Cat. #V13242) was purchased from ThermoFisher Scientific (Waltham, MA, USA). CaspaTag^TM^ Caspase-3,7 Situ Assay Kit (Cat. # APT403) was purchased from Sigma Aldrich (Burlington, MA, USA). Antibodies specific to Phospho-BRAF (Ser445) (Cat. #2696) (1:1000), RAF1 (CRAF) (Cat. #53745) (1:1000), Phospho-RAF1 (Ser338) (Cat. #9427) (1:1000), GAPDH (horse radish peroxidase (HRP)-Conjugated) (Cat. #8884) (1:1000), KRAS (Cat. #53270) (1:1000), Phospho-MAP2K1/2 (MEK1/2, Ser217/221) (Cat. #9154) (1:1000), p44/42 MAPK (ERK1/2) (Cat. #4695) (1:1000), Caspase 3 (Cat. # 9662S for cleaved and full-length) (1:1000), Caspase 7 (Cat. #9492S for cleaved and full-length) (1:1000), Phospho- RPS6KA1 (p90RSK, Ser380) (Cat. #11989) (1:1000), RAC1/2/3 (Cat. #2465) (1:1000), CDC42 (Cat. #2462) (1:1000), and anti-mouse IgG, HRP-linked Antibody (Cat. #7076) (1:3000), anti-rabbit IgG, HRP-linked Antibody (Cat. #7074) (1:3000) were purchased from Cell Signaling Technology (Danvers, MA, USA).

#### Determination of effects of PCAIs on cell viability

NCI-H23 (CRL-5800) cells were cultured in complete RPMI 1640, (Genesee Scientific, San Diego, CA) supplemented with 100 U/mL streptomycin,100 μg/mL penicillin and 10% heat-inactivated fetal bovine serum (FBS) (Genesee Scientific, San Diego, CA) at 37°C in 5% CO_2_/95% humidified air. During the treatment with PCAIs (Table 1), the cells were cultured in the same media except that only 5% heat inactivated FBS was used (experimental media). The PCAIs were dissolved in acetone for the treatment of cells in culture. The final concentration of acetone introduced into the culture media during treatment with PCAIs or vehicle controls was 1% or less. To determine the PCAIs’ potential as anticancer agents, the resazurin cell viability assay was used. The cells were plated at a density of 1.0 × 10^4^ cells per well in experimental medium in 96-well tissue culture plates (Genesee Scientific, San Diego, CA, USA) and incubated for 24 h to allow them to adhere. The cells were then treated with 0–50 μM of the respective PCAIs dissolved in acetone, using an equal volume of acetone as the vehicle control. Repeat treatments were conducted 24 h later. After 48 h exposure to the PCAIs, 20 μL of resazurin (2 μg/mL) was transferred into each well and incubated for 2–3 h. Fluorescence intensities were determined with excitation set at 495 nm and emission set at 580 nm using GloMax Explorer Microplate Reader (Promega, Madison, WI, USA). Cell viability was expressed as the percentage of the fluorescence of the treated cells relative to that of the controls as previously described [29]. EC_50_ (effective concentration causing 50% change relative to the controls) values were then obtained from nonlinear regression plots of fluorescence intensities against the concentrations of the respective PCAIs using GraphPad Prism version 8.0 for Windows (San Diego, CA).

**Table 1:** PCAIs inhibit the viability of NCI-H23 cells. Cells were seeded and treated with the respective PCAIs for 48 h followed by determination of the cell viability as described in the methods section. The molecular structures of the PCAIs can be found in Tawfeeq et al. [29].

Compound*	EC_50_ (μM) NCI-H23
**NSL-YHJ-096**	18 ± 0.9
**NSL-YHJ-31 CONTROL**	>50 ± 2.4
**NSL-YHJ-2-62 CONTROL**	>50 ± 0.9
**NSL-YHJ-2-56 CONTROL**	>50 ± 2.0
**NSL-BA-055**	2.0 ± 0.07
**NSL-YHJ-2-40**	3.2 ± 0.02
**NSL-YHJ-2-37**	3.1 ± 0.4
**NSL-YHJ-2-35**	1.9 ± 0.1
**NSL-YHJ-2-27**	2.7 ± 0.4
**NSL-YHJ-2-44**	4.4 ± 0.2
**NSL-YHJ-2-45**	4.0 ± 0.2
**NSL-YHJ-2-46**	3.3 ± 1.0
**NSL-YHJ-2-47**	6.3 ± 1.7
**NSL-YHJ-2-48**	7.1 ± 0.1
**NSL-YHJ-2-67**	7.2 ± 0.2
**NSL-YHJ-2-89**	2.2 ± 0.02
**NSL-YHJ-2-84**	>50 ± 3.1

#### Effects of PCAIs on protein and protein phosphorylation levels

Cells were plated at a density of 7 × 10^5^ cells in 60.8 cm^2^ tissue culture dishes (Genesee Scientific, San Diego, CA) in experimental media and incubated for 24 h. The experimental media were then replaced with fresh experimental medium, and the cells treated with 0–5 μM PCAIs dissolved in 20 μL of acetone per 10 mL of culture medium (2 μg/mL or 0.2% acetone final concentration). After 24 h, treatments were repeated followed by incubation for a further 24 h. The cells were washed three times with 5 mL of 1× PBS and lysed with 200 μL RIPA buffer (Genesee Scientific, San Diego, CA) supplemented with 0.1% v/v protease/phosphatase inhibitors cocktail (Cell Signaling Technology, Danvers, MA). Quick Start™ Bradford assay was used to determine the amount of protein in the harvested lysates. Cell lysates containing 30 μg protein were mixed with 50 μL of 4 x XT sample buffer and 10 μL of 20 x XT reducing agent (Bio-Rad, Hercules, CA) and boiled for 5 min. The prepared lysates were separated by SDS-PAGE on 4–12% Criterion™ XT Bis-Tris protein gels and transferred onto Trans-Blot turbo midi 0.2 μm nitrocellulose membranes (Bio-Rad, Hercules, CA). Membranes were blocked for 1 h at room temperature with OneBlock™ western-CL blocking buffer (Genesee Scientific, San Diego, CA). After blocking, the membranes were incubated overnight at 4°C in fresh blocking buffer containing the respective monoclonal antibodies against the target proteins. Membranes were then washed 3–5 times with 5–10 mL of 1x Tris buffer saline Tween-20 (TBST) and incubated with HRP-linked anti-rabbit or anti-mouse antibodies at room temperature for 2 h. Immunoreactive bands were visualized using Radiance Plus (Azure Biosystems, Dublin, CA) ECL reagents per the manufacturer’s recommendations using the ChemiDoc XRS+ System (Bio-Rad, Hercules CA). Protein or protein phosphorylation levels as judged by the chemiluminescence intensities were quantified using Image Lab 6.0 (Bio-Rad, Hercules CA) and normalized against the corresponding GAPDH band intensities. The normalization was done by dividing the intensities of the target protein by those of either GAPDH for each treatment/lane and then dividing the respective ratios by those of the control lanes. The results from three independent experiments were then plotted using GraphPad Prism version 8.0 for Windows (San Diego, CA).

#### Fluorescence-activated cell sorting (FACS) analysis of PCAIs-induced apoptotic cell death

To determine the mechanism of PCAIs-induced cell death, the FITC Annexin V/ Dead Cell Apoptosis Kit was used. It involves the binding of FITC-conjugated Annexin to externalized phosphatidylserine of apoptotic cells and propidium iodide (PI) binding to the nucleic acids of necrotic cells with compromised cell membranes. Specifically, cells at a density of 5 × 10^5^ cells/dish were cultured in experimental media overnight in 60.8 cm2 tissue culture dishes. NSL-YHJ-2-27 (0–5 μM) was added at the beginning and after 24 h. At 48 h of treatment, the cells were harvested using trypsin and suspended in 15 mL of experimental media. The cells were then washed twice with 1X cold PBS, centrifuged and transferred to fresh, sterile 5 mL polypropylene round-bottom tubes (Corning Science, Tamaulipas, Mexico) using 100 μL of 1X annexin binding buffer. To label the cells, 5 μL of FITC reagent and 1 μL PI (1 mg/ml) were added and gently mixed into the suspension and incubated for 15 min while shielding from light. After incubation, 400 μL of 1X annexin binding buffer was added to each tube, mixed gently on ice and immediately analyzed using the Sony SH800 Cell Sorter with Software version 2.1.6 (Sony Biotechnology, San Jose, CA, USA). The photomultiplier settings were adjusted to detect annexin V/FITC fluorescence on the FL2 detector and PI fluorescence on the FL3 detector. The live cells of untreated control sample on the forward and side scatter plot were gated and used for data acquisition. The spillage of the green and red fluorescence signals into the FL2 and FL3 channels were compensated with the compensation wizard using unstained cells (double negative), annexin V/FITC single stained cells, and PI single stained cells. The cell events are displayed as quadrant dot plots in the log mode; the X-axis indicating the fluorescence of annexin V/FITC (green), while the Y-axis indicates PI (red). In this study, untreated (control) and treated samples were stained with both dyes and a total of 15,000 gated individual events were analyzed separately for each sample.

*Fluorescence determination of PCAIs-induced active caspase levels*. Active caspases such as caspases 3 and 7 are enzymes that play a vital role inducing apoptosis. To determine the levels of the PCAIs induced active caspase levels, the CaspaTag^TM^ caspase-3/7 *in situ* fluorescein kit was used. Following the manufacturer’s protocol, NCI-H23 (1 x 10^5^ cells/mL) were seeded onto an 8-well μ slide plate (ibidi) and incubated overnight to adhere. The complete media were removed and replaced with treatment media containing 0, 0.5, 1, 3 and 5 μM of NSL-YHJ-2-27 followed by a 24 h incubation. The treatment was repeated after 24 h followed by another 24-h incubation period for a total of 48 h exposure. The treatment media were removed and replaced with CaspaTag FLICA reagent (1:30 dilution) in experimental media. The 8-well μ slide plates (ibidi) were incubated for 1 h at 37°C under 5% CO_2_. The media were then removed, and the cells rinsed with 2 mL 1X wash buffer. The cells were analyzed using the Keyence BZ-X800 microscope. Images were captured and the mean fluorescence intensities per cell were quantified using the BZ-800 analyzer and graphed using GraphPad Prism 8.4.3

#### Effect of PCAIs on cell migration and invasion

The wound-healing method was used to determine the effects of PCAIs on cell migration. Cell culture inserts from ibidi (Martinsried, GE) were used to create a wound in wells according to the manufacturer’s protocol. Cells were seeded at a density of 2.0 × 10^5^ cells/mL on each side of the insert on a 12-well plate with serum-free media to limit excessive cell growth and to initiate a state of starvation. The plate was then incubated at 37°C/5% CO_2_ overnight to allow the cells to attach. The following day, the inserts were removed, creating a “wound” between two adherent confluent monolayers of cells. The cells were then washed once with medium, and fresh experimental medium mixed with 0–1 μM concentrations of NSL-YHJ-2-27 or NSL-YHJ-2-46 were added to the wells. Triplicate wells were prepared for each concentration and bright-field images of different areas along the “wound" in each replicate were captured at 0, 24, 48 and 72 h using the Nikon Eclipse microscope. The number of cells that migrated into the “wound” area for the control and treated cells was determined using NIS-Elements AR version 4.30. Nine images per concentration and time point were quantified.

The transwell invasion assay was used to determine the PCAIs effects on cell invasion. The manufacturer’s protocol was followed using the 24-well BD Biocoat Matrigel invasion chambers and inserts (catalogue #354480) from Corning, Bedford, MA, USA. NCI-H23 cells (2.0 × 10^5^) in serum-free media were plated in T-25 and left to attach overnight. NSL-YHJ-2-27 (0–5 μM)-containing medium was added and the mixture incubated for 24 h. The 24-well BD Biocoat Matrigel invasion chambers and inserts were rehydrated using serum-free medium and incubated for two hours at 37°C. After treatment, the cells were collected and suspended at a density of 1.0 × 10^5^ cells/mL. The inserts were filled with 500 μL of the cell suspension and the wells were filled with media containing 10% FBS. The inserts containing cell suspensions were placed into the wells with medium containing 10% FBS. These were then incubated for 24 hours at 37°C in 5% CO_2_. Cotton swabs were used to wipe the inserts to remove non-invaded cells. The inserts were then stained with 1% crystal violet after fixing for 5 minutes in 4% formaldehyde in PBS. Images were captured using the Nikon TMS light microscope and quantified using the Nikon NIS-Elements software. The number of invaded cells was plotted against NSL-YHJ-2-27 concentration.

#### Effect of PCAIs on 3D spheroid cell invasion into Matrigel

NCI-H23 cells were suspended in complete medium, seeded at a density of 2.5 x10^4^ cells/mL in 96U Nunclon Sphera plates (Thermo Scientific, Waltham, MA), and incubated at 37°C/5% CO_2_ for 72 h to form spheroids. After 72 h, half of the medium was removed, and the remainder was treated with acetone (vehicle) or NSL-YHJ-2-27, or NSL-YHJ-2-46 (0–10 μM) dissolved in acetone. Matrigel (Corning, NY, 100 μL) mixed with acetone (vehicle, 2μL) or 1 to 10 μM PCAIs contained in 2μL of acetone) were added into each well and incubated at 37°C/5% CO_2_ for 30 min for the Matrigel to solidify. Images were taken and then every 24 h thereafter for 7 days using the Nikon Eclipse microscope. Time-dependent changes in Spheroid invasion areas were measured for the control and treated spheroids using NIS- Elements AR version 4.30.

#### Effect of PCAIs on actin filaments and the focal adhesion protein vinculin

Immunocytochemical analysis of the effect of PCAIs on cytoskeletal F-actin was conducted using Alexa Fluor^TM^568 phalloidin (cat# A12380). NCI-H23 (1 x 10^4^ cells/mL) were placed onto an 8-well μ slide plate (ibidi) and incubated overnight to adhere. The complete media were removed and replaced with treatment media containing 0 μM, 0.1 μM, 0.25 μM and 0.5 μM of NSL-YHJ-2-27 or NSL-YHJ-2-46 followed by 24 h incubation. After 24 h, treatment media were refreshed followed by incubation for a further 24 h. The cells were fixed with 4% formaldehyde, permeabilized with 0.5% Triton X-100 and stained using a mixture of 1X Alexa Fluor^TM^568 phalloidin (cat# A12380) and 1X Hoechst stain (DAPI). Fluorescent images of the F- actin cytoskeleton was captured using a Keyence BZ-X800 series microscope at 40X magnification. The mean area of the cells was determined using the BZ-800 analyzer and graphed using GraphPad Prism 8.4.3.

To test the effect of the PCAIs on vinculin, NCI-H23 cells were suspended in complete media, seeded at a density of 1.5 x 10^4^ in an 8-well μ slide plate (ibidi) and incubated overnight to adhere. After attachment, the complete media were removed and replaced with treatment media containing 0 μM, 0.1 μM, 0.25 μM and 0.5 μM of NSL-YHJ-2-27 or NSL- YHJ-2-46 and incubated for 24 h. Treatments were repeated after 24 h, followed by incubation for a further 24 h. The cells were fixed with 4% formaldehyde and permeabilized with 0.5% Triton X-100. These were then exposed to blocking buffer (1% BSA) solution for 30 mins on a shaker. After blocking, the cells in each well were incubated with rabbit vinculin primary antibodies (Cell Signaling Technology) at a dilution of (1:300) overnight in a cold room. Secondary anti-rabbit IgG2 Alexa Fluor 594 antibody (Cell Signaling Technology) was introduced into each well at a dilution of (1:1000) and incubated overnight in the dark at 4°C. Then, 1X Hoechst stain (DAPI) was added into each well and incubated for 5 mins. These were then washed with 1X PBS and images were captured using the Keyence BZ-X800 series microscope at 40X magnification. The mean number of vinculin punctates and mean cell area with vinculin punctates were quantified using the BZ-800 analyzer and graphed using GraphPad Prism 8.4.3.

### Statistical analysis

All results are the means ± the standard error of the mean. Unless described otherwise, One-Way ANOVA with Dunnett’s posthoc test was used to determine statistical significance. The values of each treatment group were compared to the respective controls using GraphPad Prism version 8.0 for Windows (San Diego, CA) and *p*≤0.05 was considered significant.

## Results

### PCAIs inhibit the viability of NCI-H23 lung cancer cells

**Fig 2:**
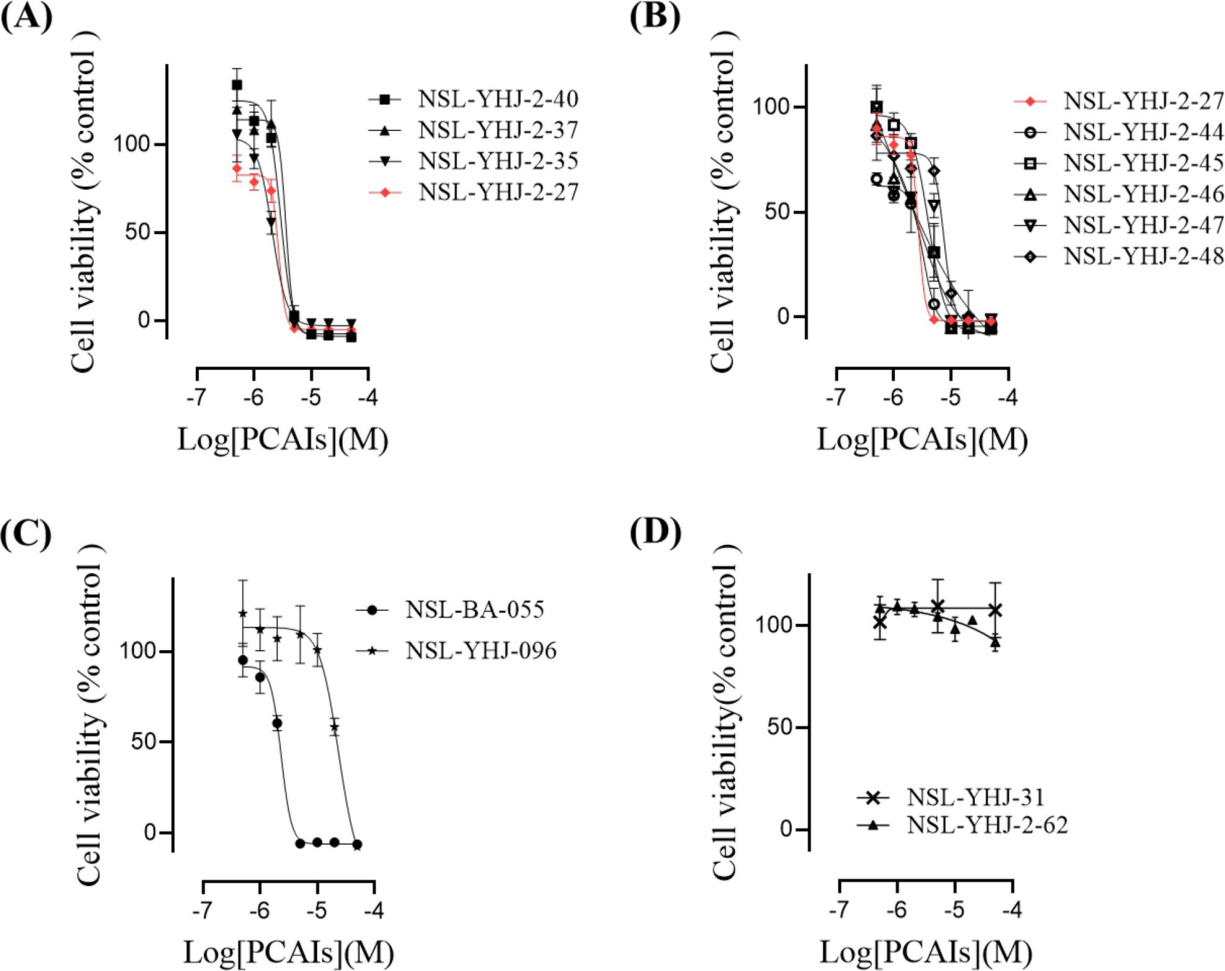
PCAIs inhibit NCI-H23 cell line viability. The effects of the PCAIs on the viability of NCI-H23 cells were determined following 48 h exposure using the resazurin reduction assay as described in the methods. Each point represents the mean ± SEM, *n* = 3. Concentration-response curves of (A) PCAIs with varying linker chain lengths, (B) PCAIs with varying cycloalkyl ring sizes, and (C) PCAIs with a free α-amino on the cysteine compared to some of the potent PCAIs analog; (D) an analog lacking an S-polyisoprenyl moiety lacked activity against cancer cell viability. The curves for the two PCAIs selected for proceeding studies are indicated in red.

As earlier stated, RAS mutations are the principal drivers in about a third of human cancer cases. Without the carboxyl terminal posttranslational modifications that are essential for their functional localization, their growth signaling will be impaired. The PCAIs were thus designed with the rationale that they will alter the secondary modifications and therefore the interaction patterns of the RAS proteins to curtail their oncogenic activities. While they were ineffective inhibitors of PMPMEase/CES1, their anticancer effects suggest that they may be competitively inhibiting RAS and related proteins from polyisoprenylation-mediated interaction sites. When a series of PCAIs were tested for their effects on the viability of NCI-H23 cells, significant decreases in cell densities and viability were observed following 48 h of treatment. As shown in Table 1 and Fig 2, the PCAIs inhibited the viability of NCI-H23 cells with EC_50_ values ranging from 1.9 to 7.2 μM. The structure-activity relationships obtained with NCI-H23 cells indicate that all the cysteinyl substituents are essential for potency. Using compound NSL-YHJ-2-27 (EC_50_ = 2.7 μM) as the reference, removal of the α-amino linker and heterocyclic substituent as in NSL-YHJ-096 (EC_50_ = 18 μM) resulted in significant loss of potency. However, decreasing the N-substituent length on the α-amino lessened the hydrophobicity of the compound while retaining high levels of potency. For instance, NSL-YHJ-2-27 with a 2-carbon linker has an EC_50_ value of 2.7 μM and compares favorably with the 6-carbon linker analog, NSL-YHJ-055, which has an EC_50_ of 2.0 μM. Loss of the S-farnesyl group as in NSL-YHJ-2-62 resulted in loss of activity, with 50 μM showing no effects on cancer cell viability. PCAIs with large N-cycloalkyl substituents as in NSL-YHJ-2-27 with a cyclooctyl moiety were more effective than those with smaller rings as in NSL-YHJ-2-48 with a cyclopropyl group. A basic group at the end of the α-amino linker appears to be essential for activity, as compound NSL-YHJ-2-84 without such a basic functional group was devoid of activity even at 50 μM. NSL-YHJ-2-27 and NSL-YHJ-2-46 were selected for further experimentation due to their potencies across cell lines, low molecular weights and low hydrophobicity.

### PCAIs stimulate BRAF but suppress CRAF (RAF1) phosphorylation

**Fig 3:**
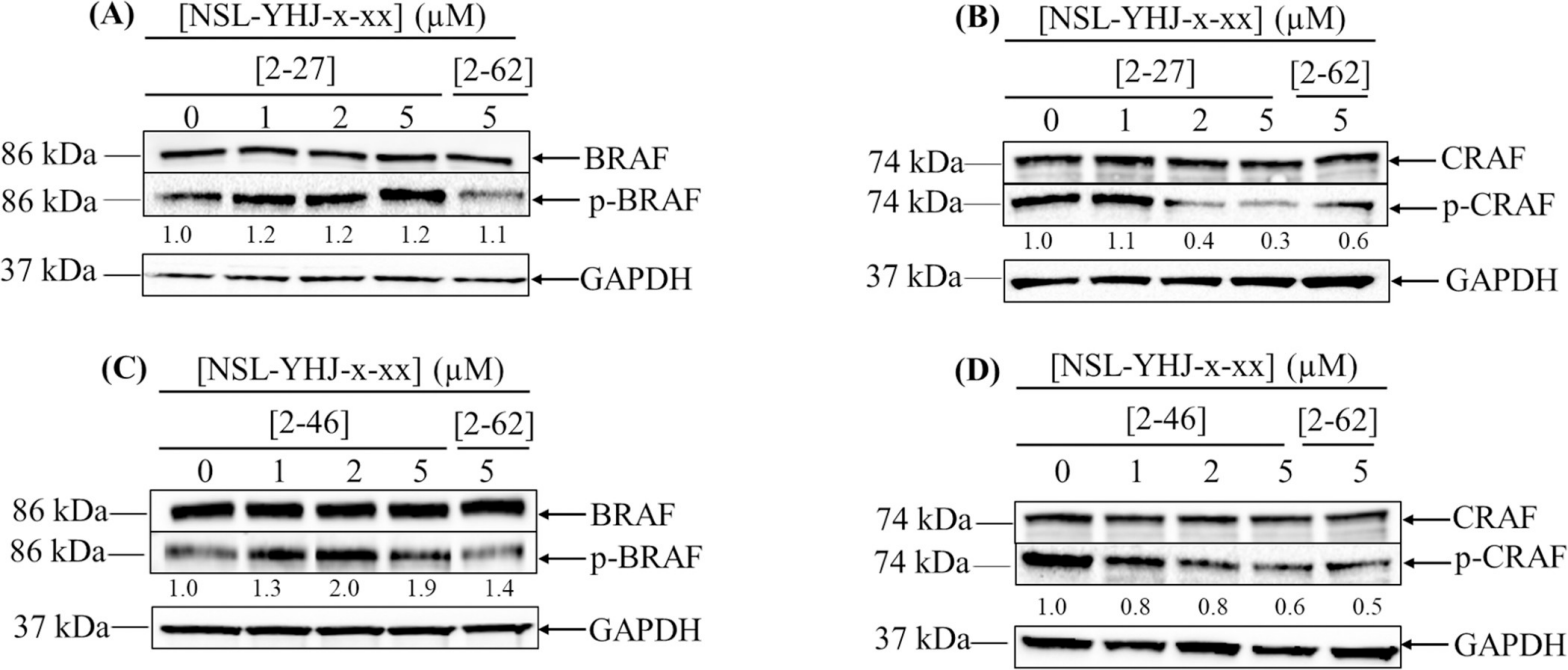
The effect of PCAIs on BRAF and RAF1 (CRAF) protein phosphorylation. NCI-H23 cells were treated for 48 h with 0−5 μM of the PCAIs (NSL-YHJ-x-xx where x-xx is 2–27 or 2–46) or 5 μM of the non-farnesylated analog, NSL-YHJ-x-xx (where x-xx is 2–62). The cells were then lysed and analyzed by western blotting to determine the levels of protein phosphorylation. Images of representative blots are shown in Fig 3A and 3B for NSL-YHJ-2-27 and Fig 3C and 3D for NSL-YHJ-2-46 treatments. Western blot images and densitometry plots of bands following quantification using Image Lab Software normalized against GAPDH and the respective total proteins, then compared to the controls. Data are representative of independent triplicate experiments. Statistical significance (*p < 0.05) was determined by One-Way ANOVA with post hoc Dunnett’s test.

RAS proteins exert strong effects on the MAPK pathway and on the functions of other G-proteins. To determine whether the action of PCAIs may involve the MAPK signaling pathway, western blot assays were conducted to determine their effects on the phosphorylation of MAPK pathway enzymes as well as on the levels of various G-proteins involved in cell viability and migration. RAF enzymes are direct downstream mediators of RAS that have been identified as a family of oncoproteins that relay extracellular messages down the MAPK pathway (Fig 1). To determine whether the PCAIs have any effects on this pathway in NCI-H23 cells, we assessed the phosphorylation levels of key proteins of the pathway following PCAIs treatment. When cells were treated with 5 μM NSL-YHJ-2-27, the phosphorylation of BRAF increased by 20 ± 0.5% (Fig 3A) while a significant 70 ± 0.2% decrease in CRAF phosphorylation was observed (Fig 3B). A similar phosphorylation trend was observed in cells treated with NSL-YHJ-2-46, in which a 90 ± 0.7% increase in BRAF phosphorylation (Fig 3C) and a 40 ± 0.4% decrease (Fig 3D) of CRAF phosphorylation were observed.

### PCAIs stimulate the phosphorylation/activation of MAP2K (MEK1/2) and MAPK (ERK1/2)

**Fig 4:**
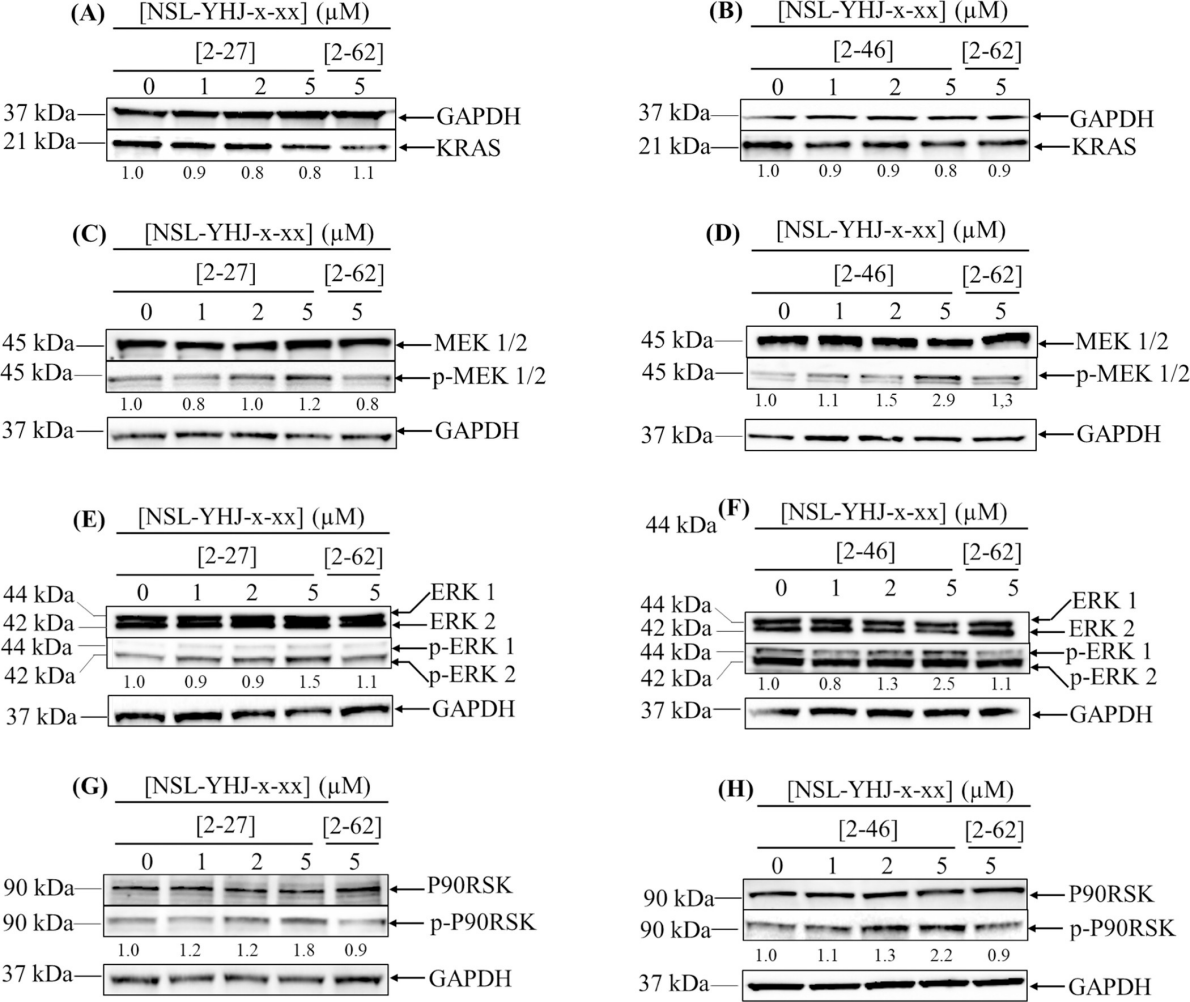
The effect of PCAIs on MAPK pathway protein phosphorylation. NCI-H23 cells were treated for 48 h with 0−5 μM of the PCAIs (NSL-YHJ-x-xx where x-xx is 2–27 or 2–46) or 5 μM of its non-farnesylated control analog, NSL-YHJ-x-xx (where x-xx is 2–62). The cells were then lysed and analyzed by western blotting to determine the KRAS levels (A and B) and phosphorylation of the MAPK pathway enzymes, MAP2K1/2 (MEK1/2) (C and D), p44/42 MAPK1/3 (p44/42 ERK1/2) (E and F) and RPS6KA1 (p90RSK) (G and H). Representative blots are shown. Western blot images and densitometry plots of bands following quantification using ImageJ Lab Software normalized against GAPDH and the respective total proteins, with the controls are to the right of the respective images. Data are representative of individual replicated independent experiments (n = 3). Statistical significance relative to the controls (*p < 0.05, **p < 0.01, and ***p< 0.001) was determined by One-Way ANOVA with post hoc Dunnett’s test.

To further our understanding of the effects of PCAIs on the NCI-H23 lung cancer cells, we probed for proteins further downstream of KRAS by western blotting (Fig 4). Significant activation of phosphorylated proteins in the MAPK pathway was observed when the NCI-H23 cells were treated with 1, 2, and 5 μM of the respective PCAIs for 48 h. The results show that NSL-YHJ-2-27 induced significant increases in the phosphorylation of MAP2K1/2 (MEK1/2), MAPK1/3 (ERK1/2), and RPS6KA1 (p90RSK) by 20 ± 0.3, 50 ± 0.2, and 80 ± 0.2%, respectively (Fig 4C–4G). Similarly, treatment of the cells with NSL-YHJ-2-46 showed significant increases in the phosphorylation of these proteins by 190 ± 0.5, 150 ± 0.6, and 120 ± 0.8%, respectively (Fig 4D–4I). Although counterintuitive, significantly increased activation of MAPK pathway proteins has been reported to spur cell death [30,31].

### PCAIs induced apoptosis in NCI-H23 cells involve the cleavage activation of apoptotic caspases

**Fig 5:**
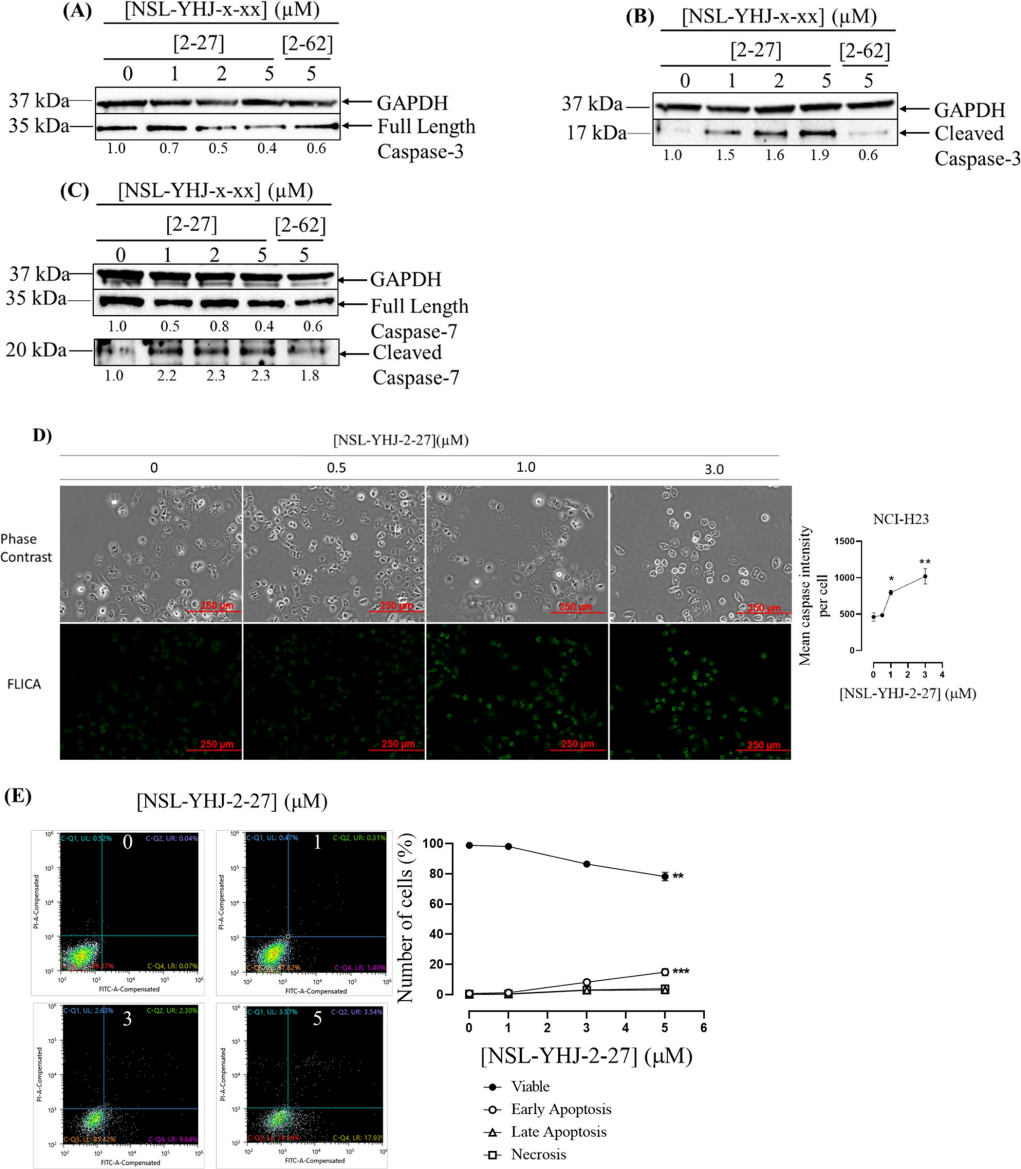
PCAIs induce apoptosis in NCI-H23 cells. (A-C) NCI-H23 cells were treated for 48 h with 0−5 μM PCAIs (NSL-YHJ-2-27) or 5 μM of the non-farnesylated control analog, NSL-YHJ-2-62. The cells were then lysed and analyzed by western blotting for the levels of the respective proteins. Representative blots are shown. Image Lab Software was used to quantify the chemiluminescence images that were then normalized against GAPDH and controls and then plotted against the PCAIs concentrations. (D). After NSL-YHJ-2-27 (0–3 μM) treatments on an 8-well μ slide plate (ibidi), the cells were treated with CaspaTag^TM^ caspase-3/7 reagent. The images were captured using the Keyence BZ-X800 fluorescence microscope and analyzed using Keyence BZ-X800 analyzer software. Mean fluorescence intensities per cell against the respective concentrations were plotted. (E) Cells after 48-h treatment with the indicated concentrations of NSL-YHJ-2-27 (0, 1, 3, 5 μM) were collected, stained with annexin V/FITC and PI and analyzed on the Sony SH800 Cell Sorter. The data were analyzed using Cell Sorter Software version 2.1.6. Quadrant statistics were used to quantify the cell populations in the quadrants. The data are representative of triplicate independent experiments. Statistical significance (*p < 0.05, **p < 0.01, and ***p< 0.001) was determined by One-Way ANOVA with post hoc Dunnett’s test.

The caspase signaling cascade plays a vital role in cell apoptosis (natural programmed cell death), a process that is necessary for maintaining proper tissue cell renewal while preventing proliferation [32]. Once cleaved, the caspase proteins are activated to initiate and continue the apoptotic process. We thus set out to determine the effects of PCAIs on the apoptotic caspases, caspases 3 and 7, by western blotting. We further evaluated the PCAIs apoptotic effects using FACS (Fig 5). After treatment with NSL-YHJ-2-27, 72 ± 11 and 60 ± 9.0% depletions of the inactive full-length caspase 3 (Fig 5A) and 7 (Fig 5C) were observed while cleaved active caspase 3 (Fig 5B) and 7 (Fig 5C) levels increased significantly by 274 ± 89 and 130 ± 11%, respectively. Activation of this apoptotic pathway supports the observation of decreased cell viability. The apoptotic potential of the PCAIs is further substantiated with fluorescence microscopy detecting labeled active caspase using the CaspaTag^TM^ Caspase-3,7 *In Situ* Assay. Fluorescence intensities increased as the concentration of the PCAIs increased with NSL-YHJ-2-27 (3 μM) significantly increasing active caspase levels by 124 ± 35% (Fig 5D). At 5 μM, virtually all the cells were detached.

Furthermore, treatment with NSL-YHJ-2-27 at 1, 3, and 5 μM caused a shift in cells from the viable to early apoptosis stage by 1.2 ± 0.1 and 8.2 ± 1.4%, and 16 ± 1.4, respectively (Fig 5E). For FACS analysis, harvesting and centrifugation after PCAIs treatment is necessary to obtain cells for sorting. Many non-viable rounded and floating cells do not get pelleted during the processing steps, thereby resulting in undercounts of apoptotic and possibly necrotic cells. The apoptotic effects of the PCAIs have potential beneficial therapeutic outcomes to inhibit tumor growth and possible reduction in tumor size.

### PCAIs deplete some singly polyisoprenylated monomeric G-proteins in NCI-H23 cells

**Fig 6:**
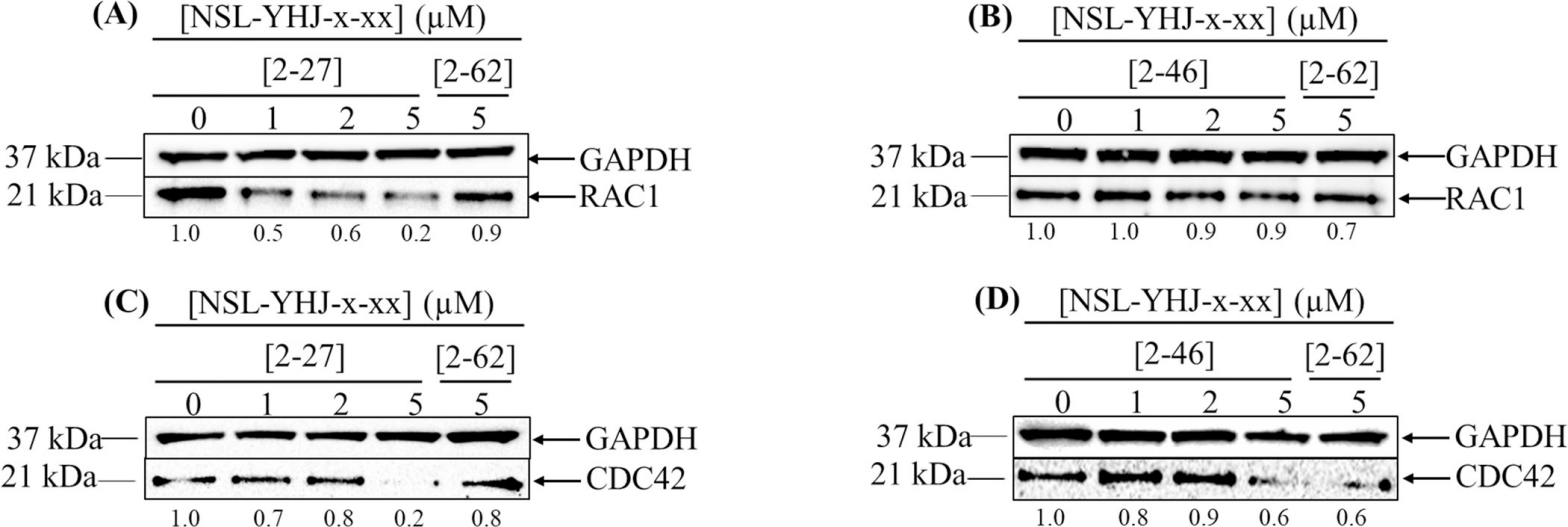
The effect of PCAIs on G-protein levels. NCI-H23 cells were treated for 48 h with 0−5 μM of the PCAIs (NSL-YHJ-2-27 or NSL-YHJ-2-46) or 5 μM of the non-farnesylated control analog, NSL-YHJ-2-62. The cells were then lysed and analyzed by western blotting for the levels of the respective proteins. Image Lab Software was used to quantify the chemiluminescence images that were then normalized against GAPDH and the controls and then plotted against the PCAIs concentrations. The data are representative of triplicate independent experiments. Statistical significance of each time points vs. t = 0 (*p < 0.05, **p < 0.01, and ***p< 0.001) was determined by One-Way ANOVA with post hoc Dunnett’s test.

Polyisoprenylation is a post-translational modification that is important for protein-protein interactions and membrane association [33]. The PCAIs were designed based on these post-translational modifications of G-proteins [34]. To determine whether the PCAIs may cause perturbations in G-protein levels, cells were treated with the PCAIs and analyzed by western blotting (Fig 6). Treatment of NCI-H23 cells with 5 μM of NSL-YHJ-2-27 for 48 h caused significant depletion of RAC1/2/3 and CDC42 and by 77 ± 5.4, and 76 ± 2.0%, respectively, while treatment with NSL-YHJ-2-46 caused a reduction in CDC42 of 36 ± 10% relative to the controls.

### PCAIs inhibit NCI-H23 cell migration and invasion and spheroid invasion into Matrigel

**Fig 7:**
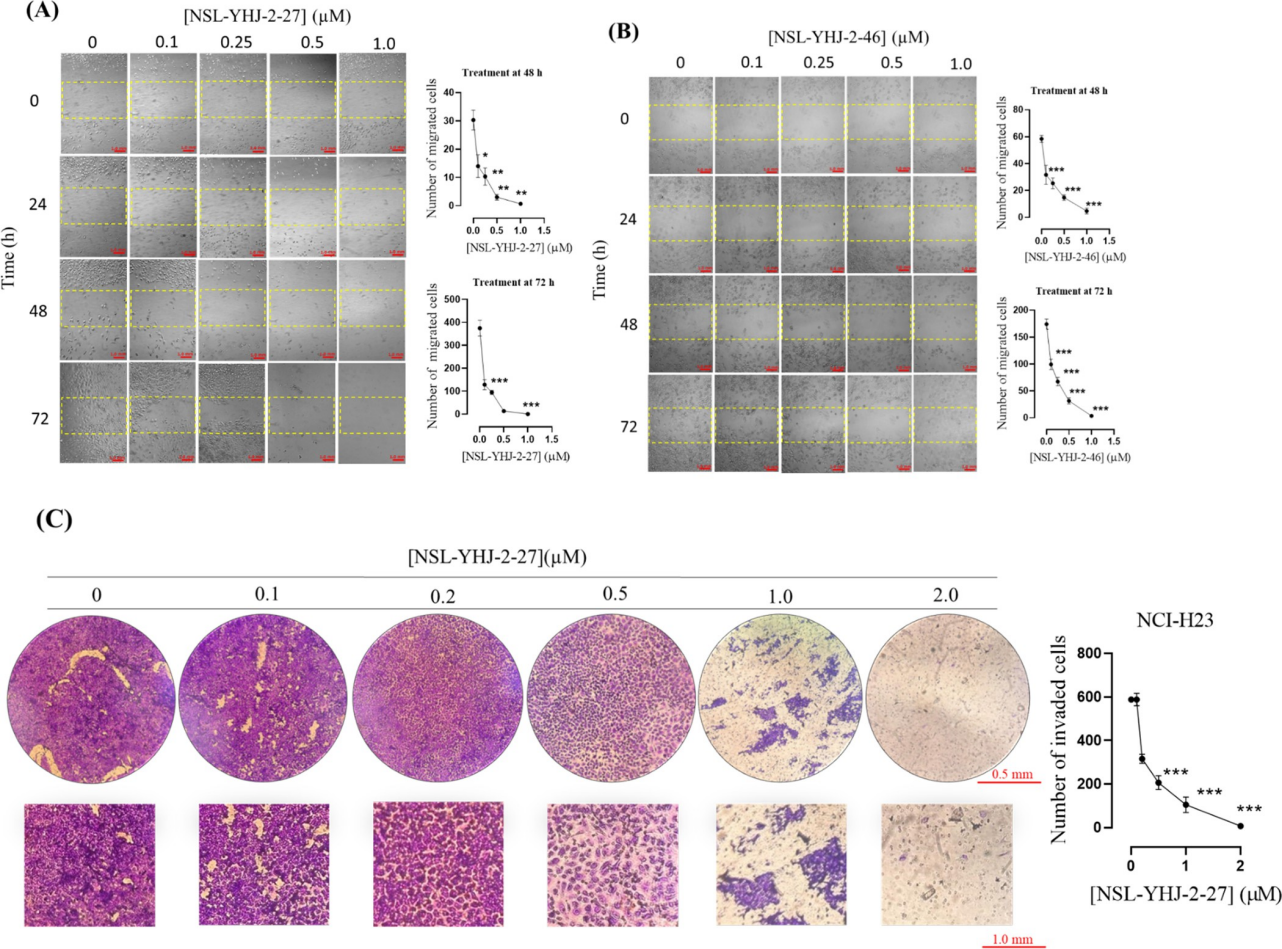
PCAIs suppress NCI-H23 cancer cell migration and invasion. (A-B) Confluent monolayers of cancer cells separated by a “wound” generated using cell culture inserts (ibidi) were treated with the indicated concentrations of NSL-YHJ-2-27 or NSL-YHJ-2-46. Closure of the wounds was monitored, and images captured at 0, 24, 48 and 72 h post-treatment using a Nikon Ti Eclipse microscope at 4X magnification. The number of cells that migrated into the wounds for nine images per treatment per time point was determined and analyzed by Two-Way ANOVA with Dunnett’s posthoc test, statistical significance (*p < 0.05, **p < 0.01, and ***p< 0.001). The difference in the number of cells for the two PCAIs is due to the difference in cell confluency at the onset. (C) Cells treated with 0–2 μM NSL-YHJ-2-27 were plated in BD Biocoat Matrigel invasion inserts, placed into chambers containing culture media and incubated for 24 h. Cells that invaded into the membranes were fixed with formaldehyde, stained with crystal violet and images captured using the Nikon TMS light microscope (Fig 7C). The number of invaded cells was determined using the NIS-Elements and analyzed by One-Way ANOVA with Dunnett’s posthoc test, statistical significance (*p < 0.05, **p < 0.01, and ***p< 0.001).

**Fig 8:**
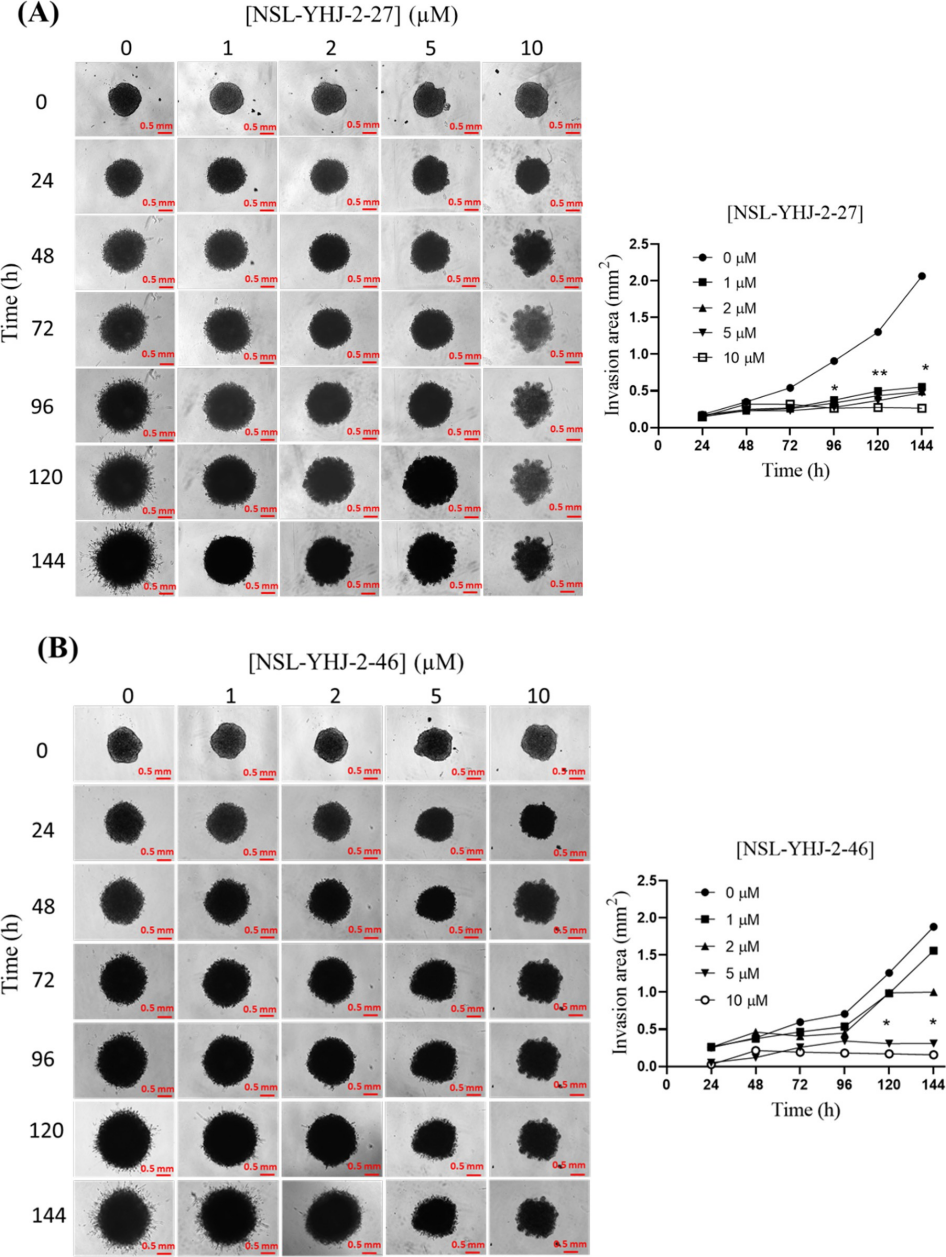
PCAIs suppress 3D NCI-H23 cell invasion. NCI-H23 preformed spheroids were treated with the indicated concentrations of either NSL-YHJ-2-27 or NSL-YHJ-2-46 dissolved in acetone (0.01 mg/ml final acetone in culture medium). PCAIs-treated Matrigel was then mixed with the spheroids and allowed for 30 mins to solidify. Images were captured at the onset (t = 0), and every 24 h for up to 144 h using the Nikon Ti Eclipse microscope at 4X magnification. Time-dependent changes in spheroid areas were measured for each treatment time point using NIS-Elements AR version 4.30. The normalized values of each treatment concentration were compared to the respective controls by Two-Way ANOVA with Dunnett’s posthoc test, statistical significance (*p < 0.05, **p < 0.01).

Metastasis happens when cancer cells break away from the primary tumor and spread through the connective tissues and basement membrane into the circulatory and lymphatic system and implant at secondary sites. The metastatic tendencies of cancers account for the lethality of most malignancies. We determined the effect of PCAIs on migration and invasion of NCI-H23 cells using the wound healing (Fig 7) and Matrigel invasion assays (Fig 8). The results reveal that 72 h exposure to NSL-YHJ-2-27 (Fig 7A) resulted in significant decreases in the number of migrated cells by 65 ± 4.7, 74 ± 2.4, and 96 ±1.3% at 0.1, 0.25, and 0.5 μM, respectively. Similarly, after a 72-h exposure of cells to 0.1, 0.25, and 0.5 μM of NSL-YHJ-2-46 (Fig 7B), cellular migration decreased significantly by 43 ± 1.2, 61 ± 1.5 and 82 ± 2.4%, respectively. The NCI-H23 monolayer cells were dead following treatment with 1 μM concentration of NSL-YHJ-2-27 and NSL-YHJ-2-46. Moreover, PCAIs at 0.5 μM and 1 μM significantly reduced the number of NCI-H23 monolayer cells that invaded BD Biocoat Matrigel inserts (Fig 7C) by 65 ± 2.5 and 82 ± 2.9%, respectively. Matrigel spheroids independently treated with 10 μM of NSL-YHJ-2-27 (Fig 8A) and NSL-YHJ-2-46 (Fig 8B) for 144 h, demonstrated a significant decrease in invasion area of NCI-H23 cells by 87 ± 0.1 and 92 ± 0.2%, respectively, relative to the controls.

### PCAIs destabilize cytoskeletal F-actin filaments

**Fig 9:**
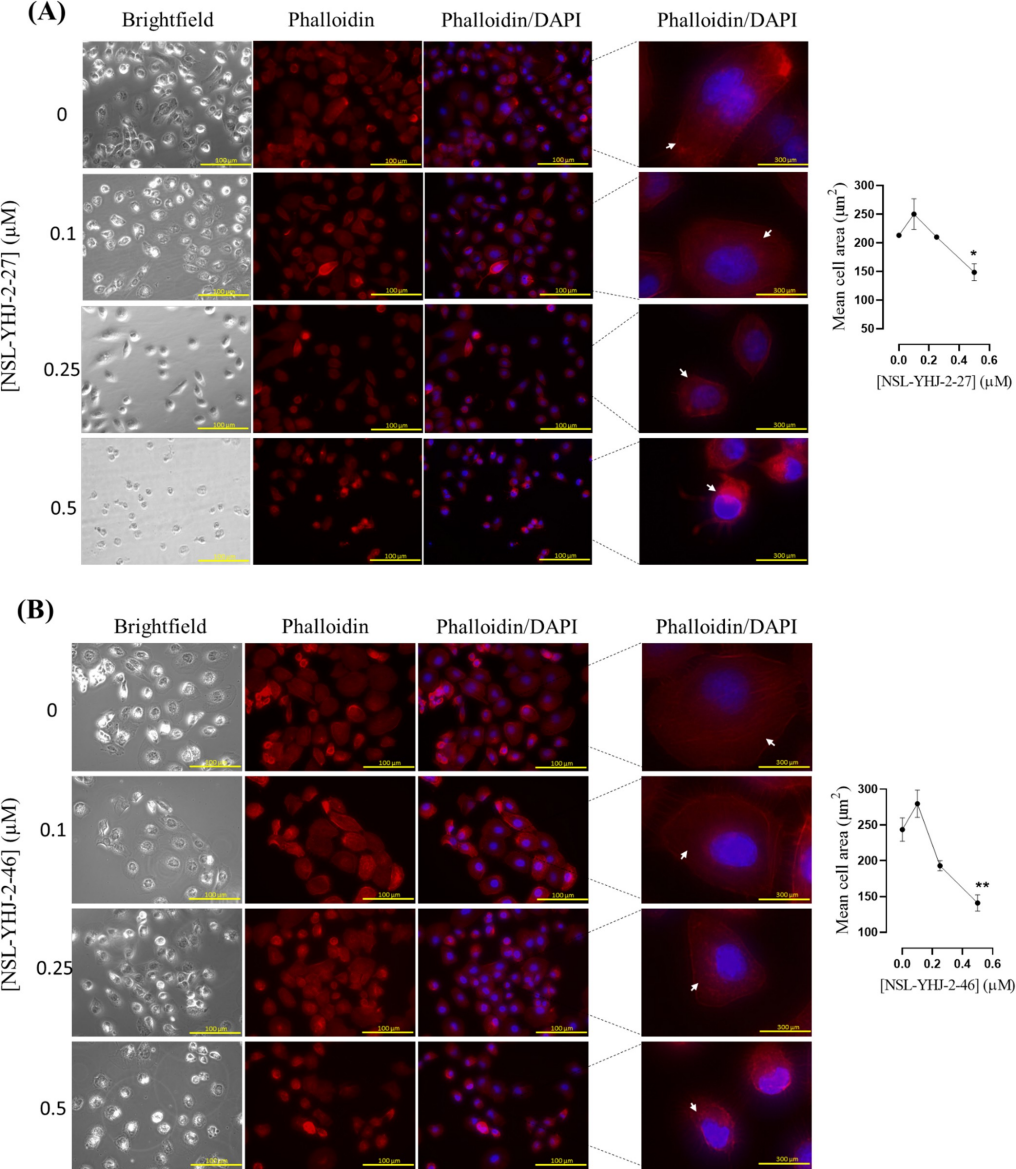
PCAIs disrupt actin filaments in NCI-H23 cells. Monolayers of NCI-H23 cells were seeded onto an 8-well μ slide plate (ibidi) and treated with the indicated concentrations of NSL-YHJ-2-27 and NSL-YHJ-2-46. The treated cells were then fixed and stained with Alexa Fluor^TM^568 phalloidin stain mixed with Hoechst stain as described in the methods. Fluorescent images of the F-actin cytoskeleton were captured with a Keyence BZ-X800 series microscope at 40X magnification. The mean cell area was quantified using the BZ-800 analyzer. The results are the means ± SEM, n = 110–300 One-Way ANOVA with Dunnett’s posthoc test was used to determine statistical significance (*p < 0.05, **p < 0.01).

**Fig 10:**
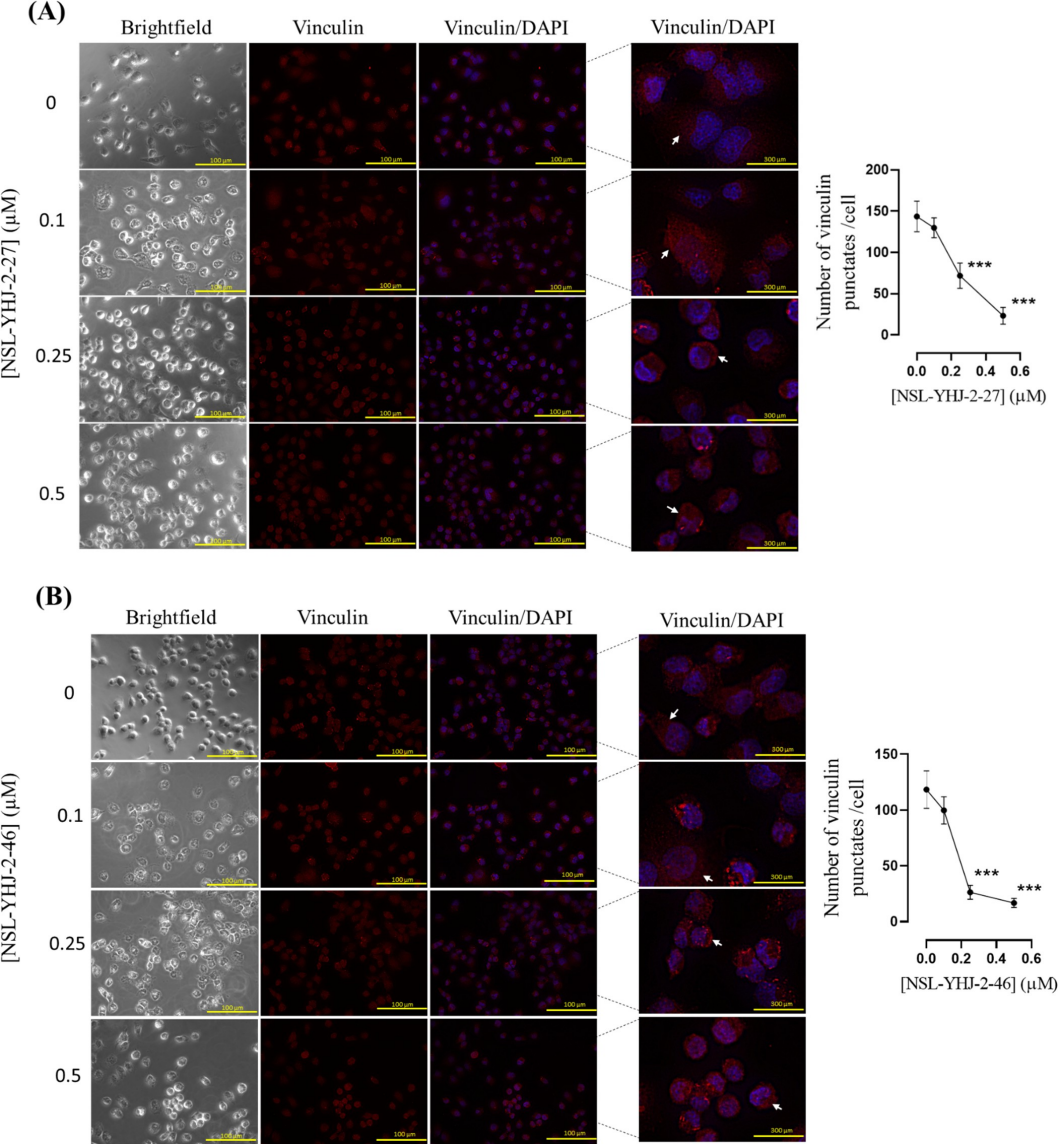
PCAIs suppress and disrupt the levels of vinculin punctates in NCI-H23 cells. Adherent NCI-H23 cells were treated with 0–0.5 μM concentrations of PCAIs for 24 h. The cells were fixed with 4% formaldehyde, permeabilized with 0.5% Triton X-100 and exposed to rabbit anti-vinculin primary antibodies at a dilution of 1:300 overnight at 4 C. After washing, these were then exposed to anti-rabbit IgG2 Alexa Fluor 594 antibody (1:1000 dilution) overnight in the dark at 4 C. Images were captured with a Keyence BZ-X800 series microscope at 40X magnification. The number of vinculin punctates and area occupied by the vinculin punctates per cell were quantified using the BZ-800 analyzer and graphed using GraphPad Prism 8.4.3. The significance was established by comparing the mean of untreated controls to the means of the different treatment groups by One-Way ANOVA with post-hoc Dunnett’s test (*p < 0.05, ** p < 0.01).

The cytoskeleton comprises microtubules, actin filaments and intermediate filaments. Together these structures provide shape and enable cell movement. Disruption of any of the cytoskeletal structures results in the loss of cell integrity and shape. We investigated the effect of the PCAIs on actin filaments in NCI-H23 cells using Alexa Fluor^TM^568 phalloidin mixed with Hoechst stain (Fig 10). Treatment of the cells with the PCAIs resulted in the reduction of the cell cytoplasm as the concentrations increased. At 0.5 μM of NSL-YHJ-2-27 (Fig 9A) and NSL-YHJ-2-46 (Fig 9B), the collapse of the active filaments resulted in significantly reduced fluorescent intensity, cell rounding and reduction in cell size. There were significant decreases in mean cell area by 30 ± 5.0 and 42 ± 0.6%, upon exposure to NSL-YHJ-2-27 (Fig 9A) and NSL-YHJ-2-46 (Fig 9B) at 0.5 μM, respectively.

### PCAIs disrupt the cellular pattern of vinculin punctates

Next, we investigated the effect of PCAIs on the actin-binding protein vinculin. Vinculin connects integrins to the F-actin cytoskeleton by attaching to α-actinin or other adapter proteins. The mean number of vinculin punctates present at focal adhesion points dropped by 84 ± 3.0% and 86 ± 0.7% after exposure to 0.5 μM of NSL-YHJ-2-27 and NSL-YHJ-2-46, respectively, when compared to the untreated controls (Fig 10). In treated cells, the vinculin punctates (arrows) appear dislodged and as the cells lose shape and become more rounded, the dislodged punctates become pressed around the cell nuclei (blue stain) compared to the more expansive distribution in untreated cells. Since vinculin is a protein that integrates actin filaments to focal adhesions, it is expected that dislodgement of the vinculin punctates would result in cell rounding while also contributing to the inhibition of cell migration and invasion. Bright field and fluorescent images of cells exposed to 0.5 μM of the PCAIs indicate that the bulk of cells remained attached yet rounded. This observation indicates the disruption and/or inhibition of the formation of focal adhesions by PCAIs.

### Discussion

Lung adenocarcinoma is the most common histological subtype of lung cancer and Black Americans show the highest lung cancer mortality. *KRAS* mutations are the most common cancer driver events in both White and Black Americans [9,35]. It is thus imperative that we identify therapies to treat *KRAS*-driven lung cancers that are effective in Black lung adenocarcinoma patients. To that end, here we have tested a series of PCAIs on NCI-H23, a lung adenocarcinoma cell line derived from a Black patient. PCAIs were previously tested on lung adenocarcinoma cell line A549 (derived from a White patient), which also carries a *KRAS* mutation [29]. Here we report that NCI-H23 cells show similar sensitivity to PCAIs. The EC_50_ values against the NCI-H23 cells were significantly lower than the EC_50_ values obtained against NCI-H1299, a non-small cell lung cancer cell line that lacks a *KRAS* mutation [29]. In the future, it would be important to confirm the efficacy of PCAIs on additional lung adenocarcinoma cell lines from all races and ethnicities that also harbor different cancer driver gene mutations.

The MAPK pathway has been associated with many cellular processes and plays important roles in cell proliferation, differentiation, apoptosis, angiogenesis, and tumor metastasis [36]. Based on the effects of PCAIs on the inhibition of cell viability, we had originally anticipated that the PCAIs would suppress the MAPK pathway. However, we observe the activation of MAP2K1/2 (MEK1/2), MAPK1/3 (ERK1/2), and RPS6KA1/p90RSK through phosphorylation, which is consistent with our previous work [34,37]. The activation of these MAPK pathway enzymes suggests that the PCAIs may directly bind to RAF isoforms to either stimulate or inhibit activation given that a polyisoprenyl-specific region exists in the RAS-binding domains of these proteins. Activated RAS stimulates the MAPK pathway from RAF culminating in cell survival [38]. It appears that the PCAIs are structurally sufficient to interact with the RAF proteins to stimulate their biochemical functions in a manner similar, at least in part, to interactions with the RAS proteins. It is uncertain why the PCAIs affect the phosphorylation of the RAF isoforms differently, whereby BRAF phosphorylation is stimulated while RAF 1 (CRAF) phosphorylation is suppressed. Considering the minimal changes in the protein levels of the mutated KRAS, the observed increase of phosphorylated BRAF and decrease of RAF1 indicate that the changes in the levels of phosphorylated RAF proteins are not due to changes in KRAS gene expression. It appears that the activation of BRAF by the PCAIs helps trigger the shrinking of cancer cells, as was elucidated in previous studies [28], since increased activation of BRAF is associated with senescence, a possible potent anticancer mechanism [39–43]. On the other hand, overexpression of RAF1 promotes cancer progression while its knockdown with siRNAs results in apoptosis, interferes with cytoskeletal activity, and dysregulates proliferation [41,44]. Thus, this implies that the decrease in the level of phosphorylated RAF1 may play a role in the suppression of cell proliferation, migration, and invasion observed in these studies. In the MAPK pathway, the observed increases in the levels of phosphorylated MAPK1/3 and RPS6KA1 are believed to be key factors in inducing cell death. Previous studies have shown that prolonged activation of MAPK1/3 displays proapoptotic effects on cells [45–47]. Meanwhile, the increase in the levels of phosphorylated RPS6KA1 could lead to the activation of RPS6KA family members, specifically, RPS6KA1 and RPS6KA3 (RSK2), which support cell proliferation and growth while RPS6KA2 (RSK3) and RPS6KA6 (RSK4) play roles in tumor suppression, cell cycle arrest, and apoptosis [48,49]. In a study conducted on human lung cancer samples, it was revealed that in metastatic lung cancer lesions, RPS6KA1 (RSK 1) was significantly reduced [50]. Furthermore, it has also been observed that metastasis is greatly increased in patients with RPS6KA1-negative lung tumors [50].

The observed significant increase in the levels of active apoptotic caspases, caspases 3 and 7, after PCAIs treatment further supports the notion that the increase in MAPK1/3 and RPS6KA1 may lead to apoptosis. The 124% increased labeling with CaspaTag^TM^ (FAM-DEVD-FMK) which is a specific irreversible inhibitor of active caspase 3/7 supports the notion that the PCAIs induce cell death via apoptosis (51). FACS analysis showed the apoptotic potential of the PCAIs on the lung cancer cells indicating that the mechanism of PCAIs-induced cell death in NCI-H23 cells is by early apoptosis. The proportion of apoptotic cells observed for the 5 μM treatment appeared to be inconsistently low relative to the observed values for the 3 μM treatment. Oversampling of viable cells due to rounded detached, likely apoptotic cells being lost during sample preparation for FACS might explain the inconsistency. Some major features of PCAIs treatment are the depletion of the focal adhesion protein vinculin, disruption of vinculin punctates and the collapse of actin filaments resulting in cell rounding [26–29]. These would explain the easy detachments of PCAIs-treated cells and subsequent inability to pellet, sort and count them.

In previous studies, PCAIs have been demonstrated to effectively induce caspase-dependent apoptosis, disrupt cytoskeleton formation, and impact angiogenesis [27,34,37,51,52]. Here, significant decreases in RAC1 and CDC42 were observed after treatment with PCAIs. These two proteins possess clusters of basic amino acid residues that bind to negatively charged phospholipid headgroups of specific membranes. The tethered positive charges found in PCAIs resemble the positive charges in the polybasic regions of G-proteins and this is believed to play a role in the ability of the PCAIs to uncouple these proteins from their polyisoprenyl-dependent interactions.

The hypothesized mechanism of action of the PCAIs of disrupting the interaction of G-proteins was based on their structural similarities to the G-proteins’ post-translational modifications (PTMs) involving single polyisoprenyl moieties [34]. Activation and functioning of a majority of small GTPases are dependent on the PTMs pathway, which is vital for membrane binding and localization of the proteins [53,54]. These modifications allow for interaction between GTPases and their upstream activators and downstream effectors in various signaling pathways, a process facilitated by the proteins’ association with the inner surfaces of plasma membranes [53]. For example, RAS recruitment of RAF to membranes is dependent on the PTMs [55]. Like the PTMs of RAC1 and CDC42, PCAIs also have a single C-terminal polyisoprenylated cysteine [56,57]. As members of the Rho GTPase family, RAC1 and CDC42 play an essential role in regulating cell division and actin cytoskeletal rearrangements [58,59]. Overexpression of these proteins is linked to cell invasion and migration, which are both central in the process of metastasis [58]. Although these two G-proteins were not affected upon treatment with NSL-YHJ-2-46, we observed significant suppression when treated with the generally more potent NSL-YHJ-2-27. The decrease in the levels of RAC1 and CDC42 coincided with the observed inhibition of cell migration and invasion upon PCAIs treatment, thereby emphasizing the potential benefits of PCAIs against metastasis. Furthermore, the observed disruption of actin filaments and vinculin punctates and the associated cell shrinkage and rounding explains the PCAIs’ ability to stop cell migration and invasion as the cells’ ability to assume various shapes, move and invade is dependent on robust cytoskeletal and focal adhesion structures. Filamentous actin is integral to the formation of lamellipodia and filopodia and together with the focal adhesion structures at their tips are essential components of the cell migratory and invasion components of the cell [27,28]. The development of filopodia and lamellipodia is a closely regulated process that is governed by such polyisoprenylated proteins as RAC1 and CDC42. Our observations that PCAIs alter F-actin organization imply that PCAIs may be acting by altering the activities of these Rho GTPases since the levels of RAC1 and CDC42 were indeed noticeably reduced in cells treated with the PCAIs. This is consistent with results of PCAIs treatment of other cancer cell lines [60].

Taken together, we propose that the activation of BRAF and MAPK1/3 affects the growth of the cancer cells by inducing cell death, while the decrease in the level of phosphorylated RAF1 and the activation of RPS6KA1 are both important in inhibiting cell migration, thereby antagonizing the spread of cancer. Furthermore, the observed decrease in the levels of RAC1 and CDC42 and the inhibitory effect on the migration and invasion of cells upon treatment with the PCAIs underline the potential of these compounds as anticancer therapeutic agents. Studies to further decipher the mechanisms and direct pharmacological targets of PCAIs are being conducted. In order to better understand potential racial and ethnic differences in response to PCAIs treatments, future work will also employ a range of lung adenocarcinoma cell lines with unique KRAS mutations especially from Blacks to determine whether the responses to PCAIs vary with different KRAS mutations.

## Supporting information

S1 Raw images The original uncropped blots for all westerns in the paper. (TIFF)

S1 Fig PCAIs inhibit NCI-H23 cell line viability. (TIFF)

S2 Fig PCAIs induce apoptosis in NCI-H23 cells. (TIFF)

S3 Fig PCAIs suppress NCI-H23 cancer cell migration. (TIFF)

S4 Fig PCAIs suppress 3D NCI-H23 cell invasion. (TIFF)

S5 Fig PCAIs disrupt actin filaments in NCI-H23 cells. (TIFF)

S6 Fig PCAIs suppress and disrupt the levels of vinculin punctuates in NCI-H23 cells. (TIFF)
