# Supplementary figures and images for: Treatment of a mutant KRAS lung cancer cell line with polyisoprenylated cysteinyl amide inhibitors activates the MAPK pathway, inhibits cell migration and induces apoptosis
Source: PLoS One. 2024 Oct 22;19(10):e0312563. doi: 10.1371/journal.pone.0312563 (PMC11495567; doi:10.1371/journal.pone.0312563)

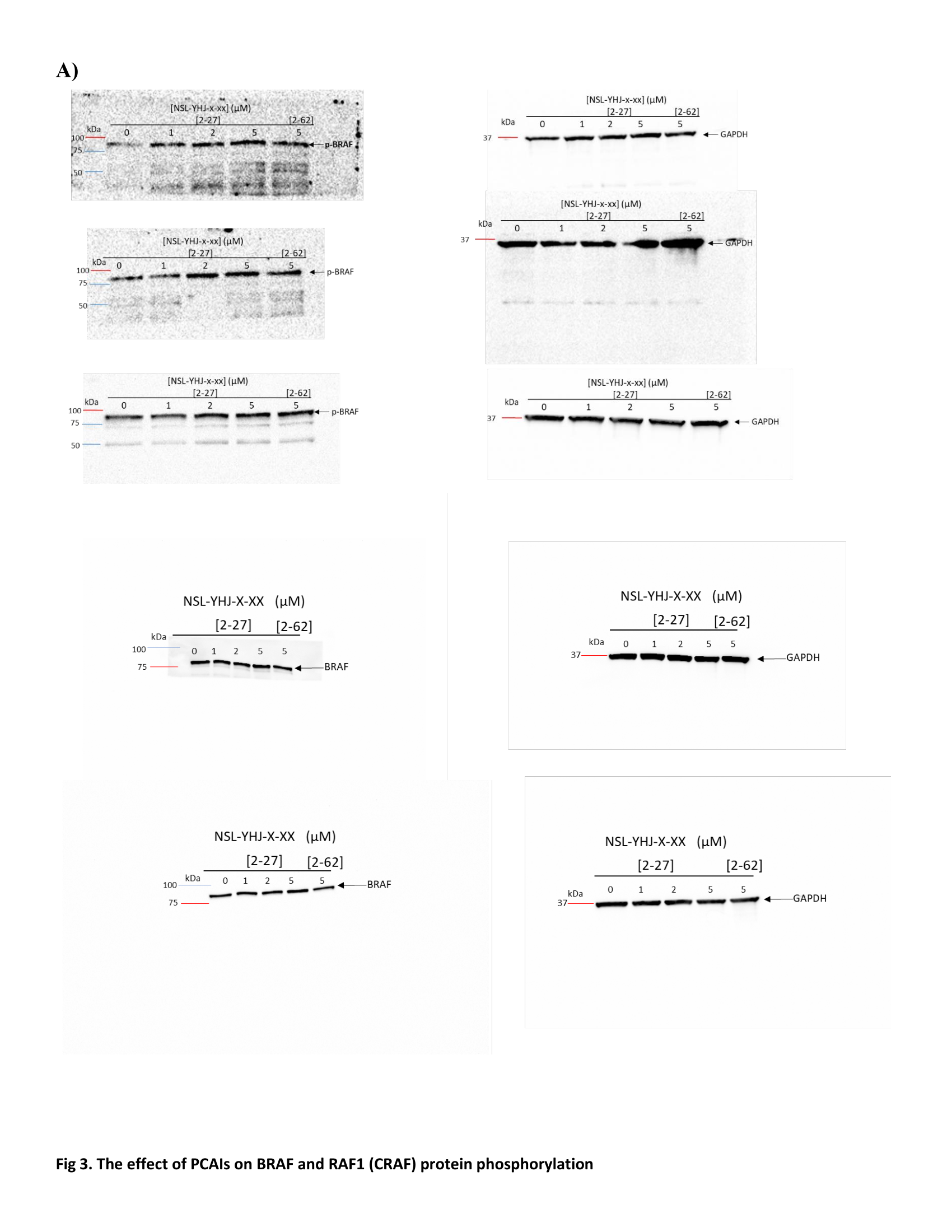

Supplement: S1 Raw images — (TIFF) [file pone.0312563.s001.tiff]

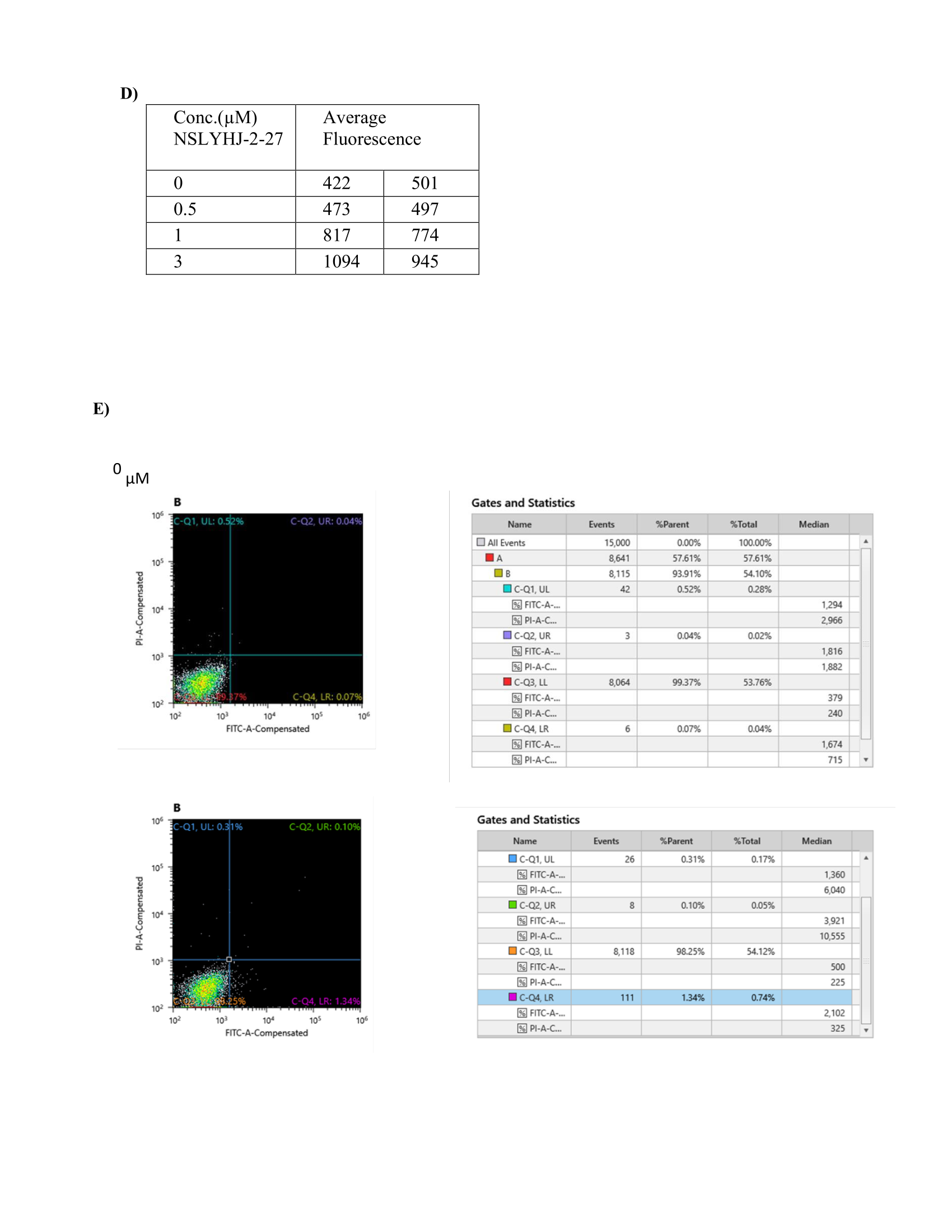

Supplement: S2 Fig — (TIFF) [file pone.0312563.s003.tiff]
